# Double-Stranded RNA Derived from Lactic Acid Bacteria Augments Th1 Immunity *via* Interferon-β from Human Dendritic Cells

**DOI:** 10.3389/fimmu.2018.00027

**Published:** 2018-01-23

**Authors:** Tadaomi Kawashima, Naho Ikari, Yohei Watanabe, Yoshiro Kubota, Sachiyo Yoshio, Tatsuya Kanto, Shinichiro Motohashi, Naoki Shimojo, Noriko M. Tsuji

**Affiliations:** ^1^Research and Development Division, Kikkoman Corporation, Chiba, Japan; ^2^Biomedical Research Institute, National Institute for Advanced Industrial Science and Technology (AIST), Tsukuba, Japan; ^3^Kikkoman General Hospital, Kikkoman Corporation, Chiba, Japan; ^4^The Research Center for Hepatitis and Immunology, National Center for Global Health and Medicine, Chiba, Japan; ^5^Department of Medical Immunology, Graduate School of Medicine, Chiba University, Chiba, Japan; ^6^Department of Pediatrics, Graduate School of Medicine, Chiba University, Chiba, Japan

**Keywords:** lactic acid bacteria, double-stranded RNA, human dendritic cells, interferon-β, interleukin-12, Th1

## Abstract

Lactic acid bacteria (LAB) are one of the major commensal species in the small intestine and known for contributing to maintenance of protective immunity and immune homeostasis. However, currently there has been no evidence regarding the cellular mechanisms involved in the probiotic effects of LAB on human immune cells. Here, we demonstrated that LAB double-stranded RNA (dsRNA) triggered interferon-β (IFN-β) production by human dendritic cells (DCs), which activated IFN-γ-producing T cells. Interleukin-12 (IL-12) secretion from human DCs in response to LAB was abrogated by depletion of bacterial dsRNA, and was attenuated by neutralizing IFN-β, indicating LAB dsRNA primarily activated the IFN-β/IL-12 pathway. Moreover, the induction of IL-12 secretion from DCs by LAB was abolished by the inhibition of endosomal acidification, confirming the critical role of the endosomal digestion of LAB. In a coculture of human naïve CD4^+^ T cells and BDCA1^+^ DCs, DCs stimulated with LAB containing dsRNA induced IFN-γ-producing T cells. These results indicate that human DCs activated by LAB enhance Th1 immunity depending on IFN-β secretion in response to bacterial dsRNA.

## Introduction

Exposure to bacterial or viral components is critical for the functional maturation of host immunity, including innate and acquired cell populations such as IFN-γ-producing Th1 cells and anti-inflammatory regulatory T cells. Therefore, microorganisms in the intestine are essential for the development of each cellular mechanism, and suppress aberrant Th2 responses at the same time (1–5). Allergic diseases or pollinosis caused by excessive Th2 immune responses may be improved by the induction of Th1 immunity, or by the anti-inflammatory effects of regulatory T cells (6–9).

The recognition of a variety of components from microorganisms by innate immune receptors triggers robust immune responses such as cytokine production (10). Toll-like receptors (TLRs) play a critical role in the recognition of structurally conserved bacterial and viral components, termed pathogen-associated molecular patterns, and signal transduction *via* TLRs induces rapid anti-infectious responses and sequentially promotes the development of acquired immunity, resulting in the maintenance of long-term homeostatic protective immunity (11–13). TLR2 and TLR4 recognize cell wall components of bacteria, while TLR3/8/9 recognize nucleic acids in endosomes (10, 14). In humans, two subsets of myeloid dendritic cells (DCs), BDCA1^+^ DCs (mDC1) and BDCA3^+^ DCs (mDC2), and plasmacytoid DCs (pDCs) are present in peripheral blood mononuclear cells (PBMCs), and mDC1 and pDCs are more abundant compared with mDC2 among these subsets (15–17). mDC1 expressing a variety of TLRs secrete high levels of interleukin-12 (IL-12), while mDC2 expressing high levels of TLR3 secrete IFN-λ, a type III IFN (18). pDCs express TLR7 and TLR9, and robustly secrete IFN-α in response to viral infection (19–21).

Lactic acid bacteria (LAB) are a major microbial species in the small intestine, and are often utilized for fermented food to prolong the preservation period and produce a variety of flavors (22, 23). Probiotic strains of LAB exert immunomodulatory effects, such as anti-infection, anti-allergy, or anti-inflammation in humans and experimental animals (24–28). Recently, it has been reported that endosomal recognition of ssRNA in *Lactococcus lactis* contributes to its allergy-protective effects (29). We previously discovered that LAB contain a large amount of double-stranded RNA (dsRNA) compared with pathogenic bacteria and can induce TLR3-mediated IFN-β production (28). Here, we elucidate the immunomodulatory role of bacterial dsRNA that induce IFN-β and IL-12 production from human DCs. Furthermore, how bacterial dsRNA promotes Th1 differentiation and that the induction of IFN-γ-producing T cells is partially dependent on IFN-β.

## Materials and Methods

### Preparation of LAB

Lactic acid bacteria were purchased from the Japan Collection of Microorganisms (JCM) or isolated from fermented foods (Table S1 in Supplementary Material). *Pediococcus acidilactici* strain K15, *Lactobacillus plantarum* ATCC14197^T^, *Lactobacillus pentosus* ATCC8041^T^, and *Lactococcus lactis* subsp. *lactis* ATCC19435^T^ were cultured at 30°C for 24 h in MRS broth (BD). *Lactobacillus delbrueckii* subsp. *bulgaricu*s ATCC11842^T^ and *Lactobacillus rhamnosus* ATCC53103^T^ (LGG) were cultured at 37°C for 24 h in MRS broth. Four strains of *Bacteroides* sp. were cultured at 37°C for 24 h in GAM broth (Nissui Pharmaceutical Co. Ltd.). Then, they were heat-killed at 95°C for 10 min, washed twice with saline, and suspended in saline. For the nuclease treatment of heat-killed bacteria, RNase A (from bovine pancreas, Sigma) treatment was performed under low salt conditions (10 mM Tris–HCl, pH 8.0) or high salt conditions (10 mM Tris–HCl, 0.3 M NaCl, pH 8.0) at 37°C for 2 h. RNase A-treated bacteria were washed twice with each buffer and used for subsequent experiments.

### Cell Preparation

Blood was provided from consenting, healthy donors in accordance with the Ethics Committee of Kikkoman Corporation (Chiba, Japan), and PBMCs were isolated by Ficoll-Paque PLUS (GE Healthcare). mDC1 were isolated from PBMCs by CD1c^+^ (BDCA1^+^) Dendritic Cell Isolation Kit (Miltenyi Biotec). Cell purity was >98% as assessed by staining with FITC-conjugated anti-CD11c antibody (Ab), BV421-conjugated anti-CD1c Ab and APC-conjugated anti-HLA-DR Ab (BioLegend). Naïve CD4^+^ T cells were isolated by Naïve CD4^+^ T Cell Isolation Kit II (Miltenyi Biotec). Cell purity was >98% as assessed by staining with FITC-conjugated anti-CD45RA Ab and APC-conjugated anti-CD4 Ab (BioLegend). Monocyte-derived DCs (moDCs) were prepared by culturing CD14^+^ monocytes isolated from PBMCs using CD14 Microbeads (Miltenyi Biotec) for 7 days in culture medium including IL-4 and GM-CSF (PeproTech).

### Cytokine Analysis

Peripheral blood mononuclear cells were cultured in 96-well round-bottomed plates at 5 × 10^5^ cells/well/200 μl in the presence or absence of 2 × 10^7^ bacteria for 24 h. moDCs were cultured at 1 × 10^5^ cells/well/200 μl with 2 × 10^7^ bacteria for 24 h. mDC1 were cultured at 5 × 10^4^ cells/well/200 μl with 1 × 10^7^ bacteria for 24 h. For the analysis of T cell cytokines, PBMCs were cultured at 1 × 10^5^ cells/well/250 μl with 1 × 10^7^ bacteria, plate-bound anti-CD3 Ab and IL-2 for 5 days. The level of cytokines in culture supernatants was measured by specific ELISA Sets (eBioscience).

### Flow Cytometric Analysis

Naïve CD4^+^ T cells purified from PBMCs were stimulated with mDC1 in culture medium containing anti-CD3/CD28 Dynabeads (Invitrogen) and cytokines [IL-2 under neutral conditions; and IL-2, IL-4, and anti-IFN-γ monoclonal Ab (mAb) under Th2 conditions] in the absence or presence of K15 for 4 days, and then cells were cultured in IL-2 containing medium without Dynabeads for 3 days. Thereafter, cells were suspended in PBS supplemented with 2% FBS at a concentration of 1–10 × 10^6^ cells/ml and stained with the optimal concentration of FITC-conjugated anti-CD4 Ab (eBioscience). Then, the cells were fixed in 3% paraformaldehyde buffer (BD PharMingen) for 30 min and cell membranes were permeabilized prior to staining with BV421-conjugated anti-IFN-γ Ab (BioLegend) and PE-conjugated anti-IL-4 Ab (BioLegend) for 30 min. Cells were analyzed on a FACSAria II and FlowJo software.

### Reagents

To neutralize IFN-β, anti- IFN-β mAb (BioLegend, mouse IgG1 Ab) was added at 10 µg/ml. Mouse IgG_1_ Ab (BioLegend) was used as an isotype control Ab. Poly(I:C) and LPS (both purchased from InvivoGen) were added at 50 and 10 µg/ml for each TLR ligand. To inhibit endosomal acidification, chloroquine (Sigma) was added at 5 µM.

### Analysis of Bacterial dsRNA

Nucleic acid was extracted from untreated or heat-killed bacteria. The concentration of bacterial dsRNA was determined by sandwich ELISA using mAb K1 and biotinylated mAb J2 (English and Scientific Consulting) for detection, followed by streptavidin peroxidase (Zymed) (28). The concentration of dsRNA was calculated using poly(I:C) as a standard.

### Quantitative RT-PCR

Total RNA was extracted from cells with a NucleoSpin RNA Kit (Takara) following the manufacturer’s instructions. An equal amount of total RNA (300 ng) corresponding to each priming dose was reverse-transcribed using PrimeScript RT Reagent (Takara). The cDNA obtained after reverse transcription was amplified using specific primers (purchased from Takara) and SYBR Premix Ex Taq II (Takara) following the protocols provided.

### Statistical Analysis

Error bars indicate the SD of triplicate samples of experiments in cell culture assays. The statistical significance was determined with a two-tailed Student’s *t*-test for unpaired data, and *p* values <0.05 were considered significant (*p < 0.05, **p < 0.01).

## Results

### IL-12 Secretion from PBMCs in Response to LAB-Derived dsRNA Is Partially Dependent on IFN-β

**Figure 1:**
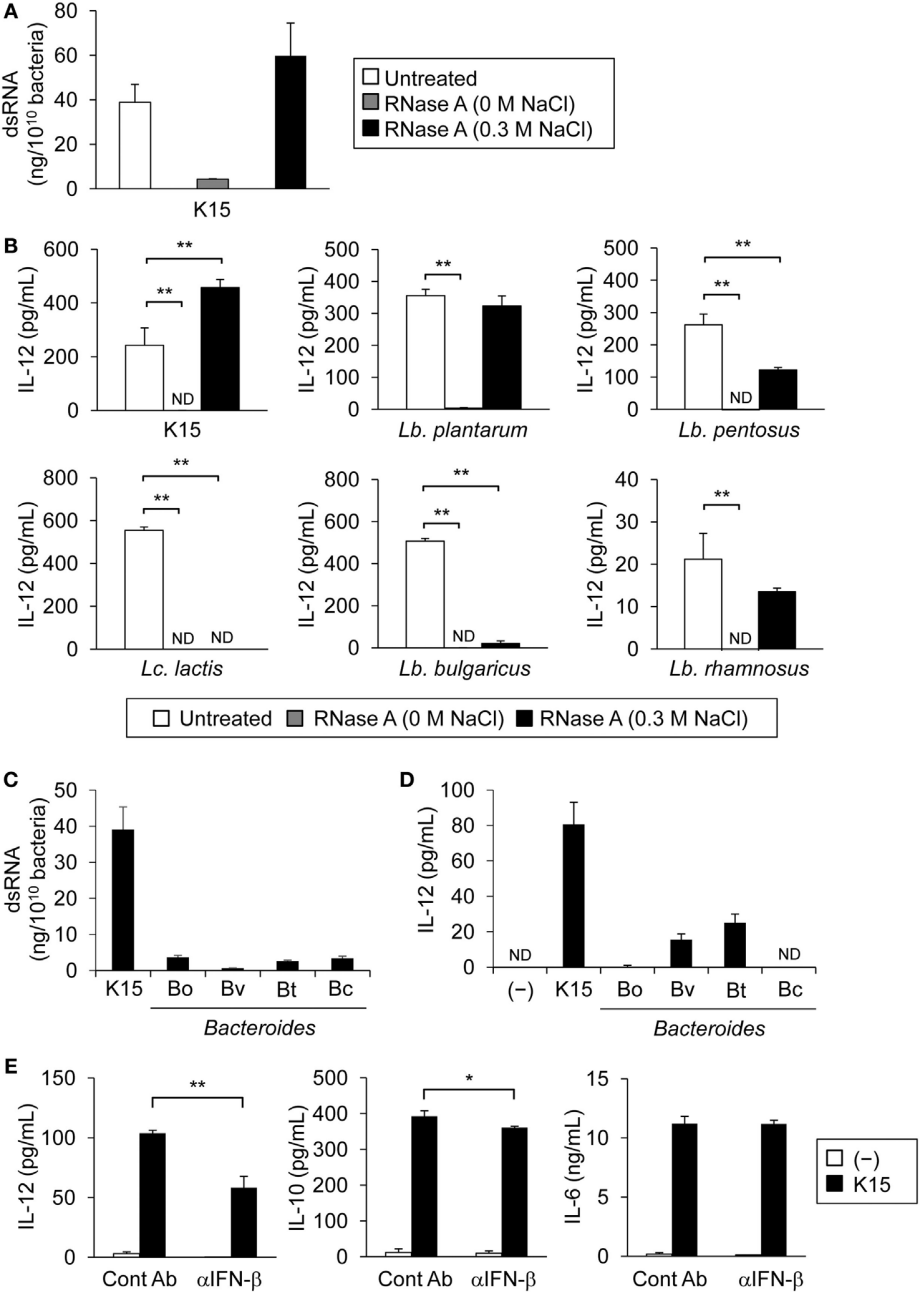
Double-stranded RNA (dsRNA) in lactic acid bacteria (LAB) induce interleukin-12 (IL-12) secretion through IFN-β from peripheral blood mononuclear cells (PBMCs). **(A)** dsRNA in *P. acidilactici* strain K15 was quantified by sandwich ELISA. Heat-killed K15 was treated with RNase A in the absence of NaCl (0 M NaCl) to degrade ssRNA and dsRNA or under 0.3 M NaCl for the degradation of ssRNA alone. Data are the mean ± SD of triplicates and are representative of two independent experiments. **(B)** PBMCs were cultured in medium alone (−) or stimulated with untreated or RNase A-treated heat-killed LAB strains for 24 h. Tested strains are described in Table 1. IL-12 concentration in culture medium was quantified by ELISA. Data are the mean ± SD of triplicates and are representative of two different donors (ND: not detected). **p < 0.01 (vs untreated, Student’s *t*-test). **(C)** dsRNA in heat-killed K15 and *Bacteroides* sp. was quantified by sandwich ELISA. Data are the mean ± SD of triplicates and are representative of two independent experiments. **(D)** PBMCs were cultured in medium alone (−) or stimulated with heat-killed K15 or *Bacteroides* sp. for 24 h. Tested strains are described in Table 1. IL-12 concentration in culture medium was quantified by ELISA. Data are the mean ± SD of triplicates and are representative of two different donors (ND: not detected). **(E)** PBMCs were cultured in medium alone (−) or stimulated with heat-killed K15 in the presence or absence of 20 µg/ml anti-human IFN-β monoclonal Ab (mAb) (αIFN-β) for 24 h. Mouse IgG1 Ab was used as the isotype control (Cont Ab). IL-12, IL-10, and IL-6 concentrations in culture medium were quantified by ELISA. Data are the mean ± SD of triplicates and are representative of two different donors. *p < 0.05, **p < 0.01 (Student’s *t*-test).

We previously reported a higher amount of dsRNA present in LAB compared with pathogenic bacteria, which contributed to anti-inflammation *via* induction of IFN-β secretion from murine DCs (28). Here, we investigated whether dsRNA in LAB triggers IFN-β production in human cells as well. RNase A digest ssRNA only in the presence of 0.3 M NaCl, but digest ssRNA and dsRNA in the absence of NaCl (30). Importantly, after treatment with RNase A with 0.3 M NaCl, the amount of dsRNA in a heat-killed LAB, *P. acidilactici* strain K15, was comparable to that in untreated K15 (Figure 1A). When PBMCs were stimulated with untreated LAB strains described in Table 1, IL-12 secretion from PBMCs was induced by various strains. However, it was abolished when both ssRNA and dsRNA were digested (Figure 1B), indicating RNA is essential for LAB to induce the secretion of IL-12. Among them, K15, *Lb. plantarum, Lb. pentosus*, and *Lb. rhamnosus* retained their ability to induce IL-12 under the condition that only ssRNA was digested. On the other hand, *Lc. lactis* and *Lb. bulgaricus* lost their ability to induce IL-12 after digestion of their ssRNA (Figure 1B; Figure S1A in Supplementary Material). These results indicate that dsRNA contained in LABs largely contributes to IL-12 induction in most of LAB strains tested while in some LAB strains ssRNA is involved in the induction of IL-12. Different effects of dsRNA and ssRNA observed among different species of LAB are brought by yet unknown mechanisms. The secretion of IL-10 and IL-6 from PBMC induced by LABs was not attenuated by digesting RNAs (Figure S1B in Supplementary Material). We also confirmed that dsRNA contained in other commensal species of bacteria, *Bacteroides*, was detected at very low level (Figure 1C), and that they induced less amount of IL-12 secretion from PBMCs compared with a representative LAB strain, K15 (Figure 1D; Figure S1C in Supplementary Material).

**Table 1:** Strain number and organism names of bacteria.

Organism name	Strain No.	Abbreviation
**Lactic acid bacteria**		
*Pediococcus acidilactici*	K15	K15
*Lactobacillus plantarum*	ATCC14197^T^	*Lb. plantarum*
*Lactobacillus pentosus*	ATCC8041^T^	*Lb. pentosus*
*Lactococcus lactis* subsp. *lactis*	ATCC19435^T^	*Lc. lactis*
*Lactobacillus delbrueckii* subsp. *bulgaricu*s	ATCC11842^T^	*Lb. bulgaricus*
*Lactobacillus rhamnosus*	ATCC53103^T^	*Lb. rhamnosus*
***Bacteroides* sp.**		
*Bacteroides ovatus*	ATCC8483^T^	Bo
*Bacteroides vulgatus*	ATCC8482^T^	Bv
*Bacteroides thetaiotaomicron*	ATCC29148^T^	Bt
*Bacteroides caccae*	ATCC43185^T^	Bc

In our previous experiments using murine cells, dsRNA in LAB uniquely stimulated innate immune system and induced IFN-β production via TLR3 pathway (28). We next evaluated the involvement of IFN-β in IL-12 secretion by human PBMCs, in response to LAB. In a coculture of PBMCs with heat-killed K15 in the presence of neutralizing Ab to IFN-β, IL-12 secretion induced by K15 was significantly reduced, whereas production of IL-10 was slightly reduced and IL-6 was not affected, respectively (Figure 1E; Figure S1D in Supplementary Material). These results indicate that IFN-β production by PBMCs in response to LAB augments IL-12 secretion.

### IL-12 Secretion from moDCs in Response to LAB-Derived dsRNA Is Partially Dependent on IFN-β

**Figure 2:**
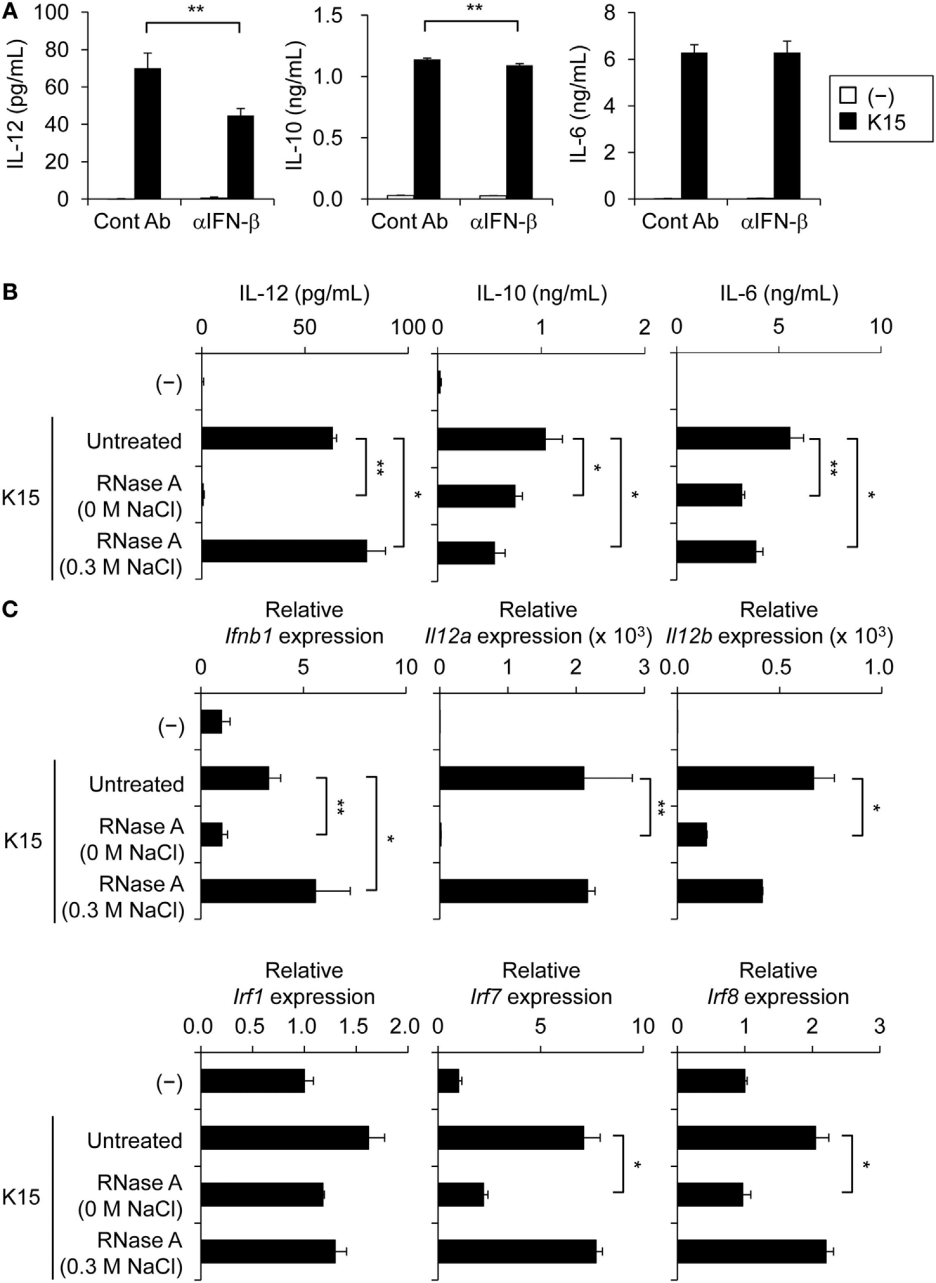
IFN-β is involved in interleukin-12 (IL-12) secretion by monocyte-derived DCs (moDCs) stimulated with bacterial double-stranded RNA (dsRNA). **(A)** moDCs were cultured in medium alone (−) or stimulated with heat-killed K15 in the presence or absence of 20 µg/ml anti-human IFN-β monoclonal Ab (mAb) (αIFN-β) for 24 h. Mouse IgG1 Ab was used as the isotype control (Cont Ab). IL-12, IL-10, and IL-6 concentrations in culture medium were quantified by ELISA. Data are the mean ± SD of triplicates and are representative of two different donors. **p < 0.01 (Student’s *t*-test). **(B)** moDCs were cultured in medium alone (−) or stimulated with untreated or RNase A-treated heat-killed K15 for 24 h. IL-12, IL-10, and IL-6 concentrations in culture medium were quantified by ELISA. Data are the mean ± SD of triplicates and are representative of two different donors. *p < 0.05, **p < 0.01 (vs untreated K15, Student’s *t*-test). **(C)** Levels of IFN-β, IL-12, and IRF mRNA expressions were determined by quantitative RT-PCR. Total RNA was extracted at 9 h after stimulation with heat-killed K15. Expression is represented as relative expression compared with unstimulated moDCs. Data are the mean ± SD of triplicates and are representative of two different donors. *p < 0.05, **p < 0.01 (vs untreated K15, Student’s *t*-test).

To evaluate the response of DCs to K15, moDCs were stimulated with K15 in the presence or absence of neutralizing Ab to IFN-β. As observed for PBMCs, IL-12 secretion from moDCs was induced by stimulation with K15 and was suppressed by IFN-β neutralization. IL-10 secretion was slightly attenuated by anti-IFN-β mAb and IL-6 was not affected (Figure 2A; Figure S2A in Supplementary Material). A previous study reported two subsets of moDCs: CD1a^+^ moDCs activated by CD40L produce a higher level of IL-12 than CD1a^−^ moDCs, whereas IL-10 secretion was higher in CD1a^−^ moDCs (31). When we stimulated each subset of moDCs with K15 after sorting (Figure S2B in Supplementary Material), both CD1a^+^ and CD1a^−^ moDCs produced IL-12 but more efficiently by CD1a^+^ cells. IL-6 and IL-10 CD1a^−^ were preferentially secreted by CD1a^−^ moDCs (Table S1 in Supplementary Material).

Next, we evaluated the involvement of dsRNA in the response of moDCs to K15. In a coculture of moDCs with RNase A-treated K15, the secretion of IL-12, but not IL-6 and IL-10, was strongly impaired by the depletion of ssRNA and dsRNA (Figure 2B; Figure S2C in Supplementary Material). The enhancement of mRNA expression of IFN-β and IL-12 by K15 stimulation was also impaired by the degradation of bacterial dsRNA (Figure 2C; Figure S2D in Supplementary Material). The expressions of genes encoding IRF families, such as IRF1, IRF7, and IRF8, which control the transcription of IL-12 (32, 33), were enhanced by K15 stimulation, and depletion of ssRNA and dsRNA impaired the induction of IRFs, especially IRF7 and IRF8 (Figure 2C; Figure S2D in Supplementary Material). Depletion of ssRNA alone in K15 did not affect IL-12 production or the mRNA expression of IRF7 and IRF8, but slightly attenuated IL-6, IL-10 production and IRF1 mRNA expression (Figures 2B,C; Figure S2C,D in Supplementary Material). These results indicate that dsRNA is essential for the induction of IFN-β secretion and IRF7/8 mRNA expression in moDCs in response to K15, and that IL-12 secretion partially depends on this IFN-β response.

### Endosomal Processes Are Essential for IL-12 Secretion in Response to LAB

TLR3, 8, and 9, which recognize nucleic acids, are localized to the endosome of DCs. As we showed in the previous study that dsRNA in LAB is recognized by endosomal TLR3 and induce IFN-β production in murine DCs (28), endosomal processes are likely to be essential for the response of human DCs to LAB. We investigated the process by stimulating moDCs with K15 in the presence of hydroxychloroquine (also called chloroquine), which blocks the signal transduction of endosomal TLRs by inhibiting endosomal acidification (34). Poly(I:C) is used as a control that stimulates endosomal TLR3, and LPS binds to TLR4 on the cell surface.

**Figure 3:**
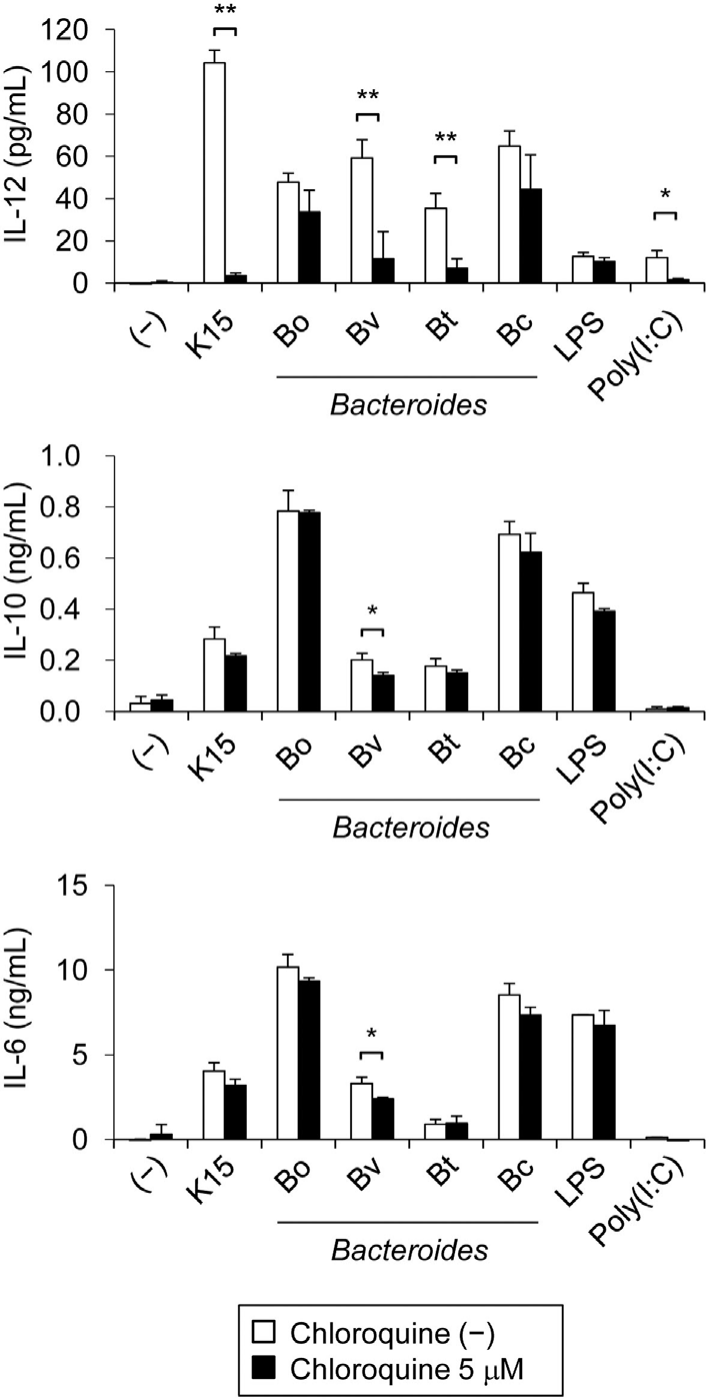
Endosomal processing is essential for interleukin-12 (IL-12) secretion by monocyte-derived DCs (moDCs) in response to lactic acid bacteria (LAB). moDCs were cultured in medium alone (−) or stimulated with heat-killed K15, *Bacteroides* sp., LPS or poly(I:C) in the presence or absence of chloroquine (5 µM) for 24 h. IL-12, IL-10, and IL-6 concentrations in culture medium were quantified by ELISA. Data are the mean ± SD of triplicates and are representative of two different donors. *p < 0.05, **p < 0.01 (Student’s *t*-test).

We observed that the production of IL-12 by moDCs in response to K15 was significantly higher than control TLR ligands, and it was totally blocked in the presence of chloroquine. The same treatment did not attenuate the production of IL-6 and IL-10 (Figure 3; Figure S3 in Supplementary Material). In stimulation with *Bacteroides* sp., IL-12 secretion was suppressed by chloroquine in some strains but this inhibitory effect was partial and strain dependent (Figure 3; Figure S3 in Supplementary Material). These results indicate that the signal transduction of endosomal TLRs is essential for the induction of IL-12 against LAB in moDCs. By contrast, the production of IL-6 and IL-10 are partially induced by RNAs but largely dependent on other recognition mechanisms.

### dsRNA in LAB Promotes the Differentiation of IFN-γ-Producing T Cells

**Figure 4:**
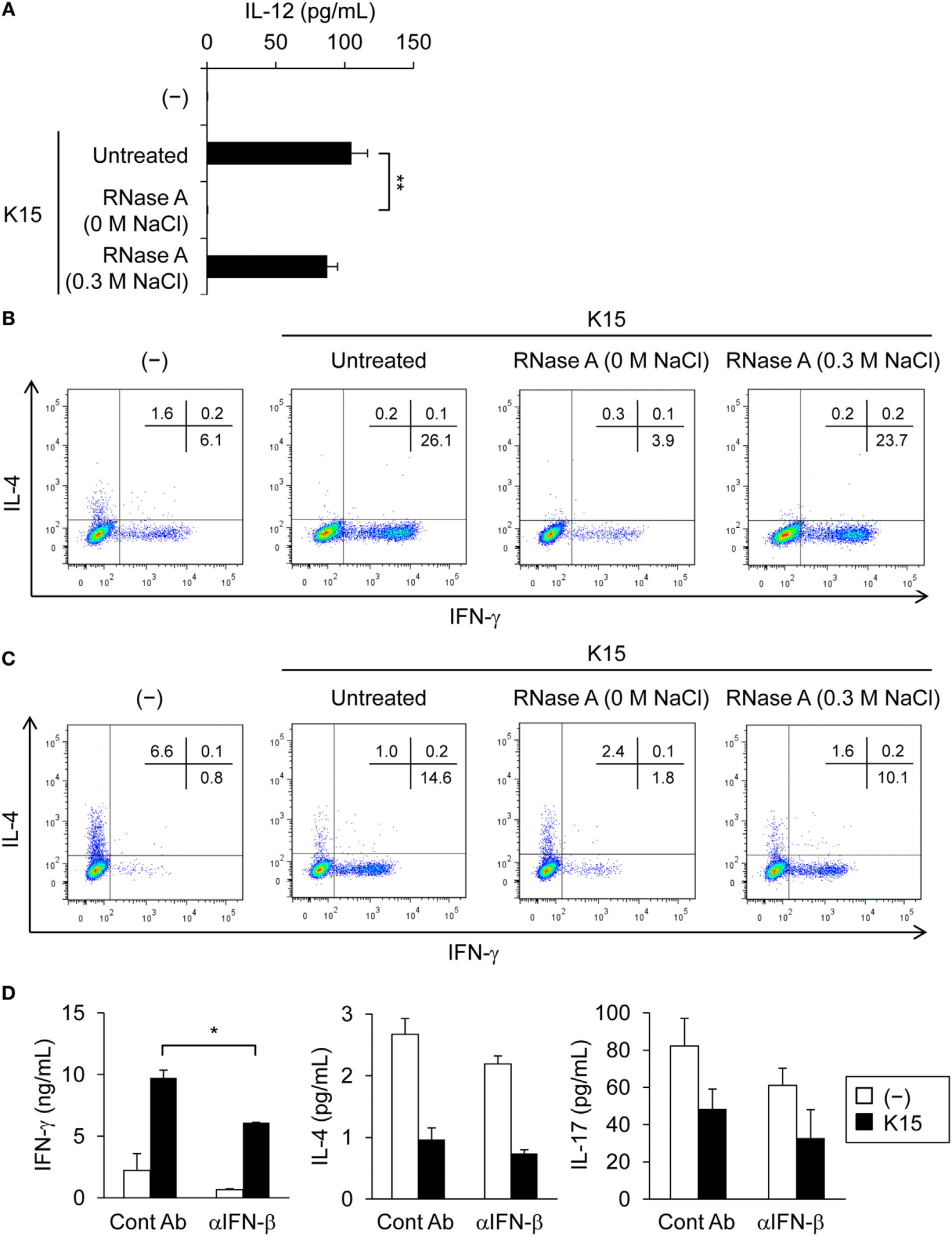
Th1 cell differentiation is induced by dendritic cells (DCs) in response to double-stranded RNA (dsRNA) in lactic acid bacteria (LAB). **(A)** BDCA1^+^ DCs (mDC1) were cultured in medium alone (−) or stimulated with untreated or RNase A-treated (0 M or 0.3 M NaCl) heat-killed K15 for 24 h. Interleukin-12 (IL-12) concentrations in culture medium were quantified by ELISA. Data are the mean ± SD of triplicates and are representative of two different donors. **p < 0.01 (vs untreated K15, Student’s *t*-test). **(B,C)** Naïve CD4^+^ T cells purified from peripheral blood mononuclear cells (PBMCs) were stimulated with mDC1 in the absence (−) or in the presence of untreated or RNase A-treated (0 M or 0.3 M NaCl) K15 under neutral conditions containing only IL-2 **(B)** or Th2 conditions containing IL-2, IL-4, and anti-IFN-γ monoclonal Ab (mAb) **(C)**. After 7 days, the percentage of cells producing cytoplasmic IFN-γ and IL-4 was determined by flow cytometry. Numbers indicate the percentage of the total cells present in each quadrant. Data are representative of two different donors. **(D)** PBMCs were cultured in medium alone (−) or stimulated with heat-killed K15 in the presence or absence of 20 µg/ml anti-human IFN-β mAb (αIFN-β) for 5 days. IFN-γ, IL-4, and IL-17 concentrations in culture medium were quantified by ELISA. Data are the mean ± SD of triplicates and are representative of two different donors. *p < 0.05 (Student’s *t*-test).

Next, we assessed the IL-12 response of myeloid DCs expressing BDCA1, termed mDC1, in PMBC to evaluate the potential activity of LAB in contact with peripheral DCs under steady-state conditions. As also indicated from Figure 1B, sorted cell population of mDC1 secreted IL-12 in response to K15, which was impaired by the degradation of ssRNA and dsRNA, but not by the degradation of ssRNA alone (Figure 4A; Figure S4A in Supplementary Material). We concluded that mDC1 should be functional antigen-presenting cells to T cells in the presence of LAB, as they secrete IL-12 in response to dsRNA in LAB similar to moDCs. We also confirmed that K15 stimulation upregulate the expression of HLA-DR and CD86 (Figures S4B,C in Supplementary Material) that is required for efficient co-stimulation to T cells upon antigen stimulation. We cocultured mDC1 and naïve CD4^+^ T cells from PBMCs in the presence of anti-CD3 Ab and IL-2, and analyzed T cell differentiation in response to K15 under neutral and Th2 conditions. K15 stimulation promoted the differentiation of IFN-γ producing T cells under both conditions, and strongly suppressed IL-4-producing T cell differentiation under Th2 conditions (Figures 4B,C; Figures S4D,E in Supplementary Material). RNase A treatment that digested dsRNA attenuated the ability of K15 to induce IFN-γ producing T cells under both conditions (Figures 4B,C; Figures S4D,E in Supplementary Material). The reduction of IFN-γ producing T cells by the digestion of dsRNA is probably caused by the attenuated IFN-β production and consequent reduction of IL-12 production.

Similar results were obtained in the whole assay of PBMCs including DCs and T cells when stimulated with K15 in the presence of anti-CD3 Ab and IL-2. K15 stimulation enhanced the secretion of IFN-γ produced by Th1 cells, and this induction was attenuated in the presence of neutralizing Abs to IFN-β (Figure 4D; Figure S4F in Supplementary Material). IL-4 and IL-17 produced by Th2 and Th17 cells, respectively, were suppressed by K15 but neutralization of IFN-β did not affect these cytokine productions (Figure 4D; Figure S4F in Supplementary Material). These results indicate that K15 induce robust IL-12 secretion from DCs *via* IFN-β production, resulting in the secretion of IFN-γ. Together these data confirm that bacterial dsRNA is involved in the recognition of K15 and contributes to the secretion of IL-12 from DCs and the differentiation of T cells toward IFN-γ producing cells, providing the molecular mechanisms to support the idea that probiotic LAB enhance Th1 immunity under steady-state conditions.

## Discussion

Here, we demonstrated that bacterial dsRNA in most strains of LAB tested was essential for IL-12 secretion from human DCs. Moreover, it was shown that IL-12 induction by stimulation with heat-killed LAB was enhanced by IFN-β through type I IFN receptors and upregulation of IRF gene transcription. Currently, there has been no evidence regarding the cellular mechanisms involved in the probiotic effects of LAB on human immune cells, we investigated the mechanisms on this issue using DCs and T cells from human peripheral blood for the first time. IL-12 and IFN-γ are major cytokines that drive T cell immunity toward Th1 response (35, 36). Other cytokines, such as IL-18 and IL-27, are also important for the efficient induction of Th1 responses *in vitro* and *in vivo* (26, 37). The enhancement of Th1 immunity by LAB, which is induced by IL-12 production from antigen-presenting cells, was observed in experimental animals (25, 26, 38, 39). Major components of the bacterial cell wall from Gram-positive bacteria enhanced IL-12 production by DCs or macrophages *via* TLR2 and/or TLR4 signaling (40, 41). However, in addition to those knowledge, we have shown that bacterial RNA is strongly involved in the induction of IL-12 production upon stimulation with LAB. A previous report indicated the involvement of bacterial ssRNA (or total RNA) in IL-12 production or type I IFNs by DCs (29, 42, 43), which, in our experiments, was observed in two species, *Lc. lactis* and *Lb. bulgaricus*, among all tested LAB. As we reported previously, a large amount of dsRNA as cellular components is a characteristic of LAB (28). Our present data now revealed that elimination of RNA abolish the production of IL-12 from PBMCs and moDCs in response to LAB. Thus RNA, especially dsRNA, is essential for the induction of IL-12. Combination effects with other molecules than IFN-β induced by the recognition of nucleic acids in LAB seems to be important to fully explain this interesting observation. Collectively, probiotic or commensal LAB seems to induce IL-12 efficiently through both the canonical pathway *via* TLR2/TLR4 and the nucleic acid sensing pathway *via* TLR3/TLR8 (28, 29, 44–46), and its synergistic effects should be uncovered in the near future.

Importantly, the IFN-β-IL-12 pathway in response to LAB was demonstrated to bridge the IL-12/IFN-γ axis in T cell immunity. Although IFN-β production is triggered by stress induced by pathogens, such as viral and bacterial infection (44–46), it was also induced by stimulation with LAB-derived dsRNA *in vitro* and *in vivo* (28). Thus, dsRNA of LAB in microbiota or fermented foods might be functional ligands for endosomal TLR3 of intestinal DCs and lead to the maintenance of T cell immune homeostasis. We also tested Bacteroides species among other commensal species which mostly reside in large intestine, and showed the clear difference from LAB. Only small amount of dsRNA was detected in Bacteroides species; therefore, the role of TLR3 seems to be marginal in the responsiveness to those commensal bacteria. This observation matches well with our previous finding that TLR3 is important to maintain the level of IFN-β in the small intestine where LAB is the major commensal bacteria species. The IFN-β-IL-12 pathway in DCs that enhance Th1 immunity seems to be unique to LAB as small intestinal commensal bacteria.

Toll-like receptor signal-mediated type I IFNs induce the expression of IFN-regulated genes including IRF7 (47, 48) and consequently contribute to the production of inflammatory cytokines, such as IL-12p35 (IL-12A) (49–51). Thus, in the regulation of IL-12p70 production, IL-12p40 (IL-12B) is induced by NF-κB activation (32) whereas IL-12p35 gene expression is induced by IRF family molecules (33, 52). We observed that the production of IL-12p70 by moDCs in response to K15 was significantly higher than control TLR ligands and it was totally blocked in the presence of chloroquine, confirming the critical role of the endosomal digestion of LAB and the recognition of their nucleic acids *in situ*. The same treatment also attenuated the production of IL-6 and IL-10 but only partially. Our data show that IRF7 mRNA expression is strongly induced by K15 and that the degradation of bacterial dsRNA suppressed IRF7 and IFN-β mRNA induction. Therefore, IFN-β production induced by bacterial dsRNA, recognized in endosome, is important for IRF7 mRNA induction and the subsequent gene transcription of IL-12p35. Since IL-12p70 was barely produced from moDCs with poly(I:C) stimulation alone at the time point of 24 h (53), it is suggested that the combination of TLR ligands in LAB contribute to induce IL-12p70, i.e., both IL-12p35 and IL-12p40, resulting in the development of Th1 immune responses. There are many reports for the immunomodulatory effects of LAB on allergic diseases, and the cellular mechanisms we showed in the present study may involve such probiotic effects.

## Ethics Statement

This study was carried out in accordance with the Ethics Committee of Kikkoman Corporation (Chiba, Japan, No. KC-RD1 and KC-RD9), and blood samples were acquired from healthy volunteers under informed written consent in compliance with the Declaration of Helsinki (2013).

## Author Contributions

TK and NI equally contributed to this work. TK, SM, NS, and NMT conceived and designed experiments. TK, NI, YW, and YK performed experiments and analyzed data. SY and TK contributed reagents/materials/analysis tools. TK, NI, and NMT wrote the paper.

## Conflict of Interest Statement

The authors declare that this research was conducted in the absence of any commercial or financial relationships that could be construed as a potential conflict of interest.
