# Supplementary material for: Double-Stranded RNA Derived from Lactic Acid Bacteria Augments Th1 Immunity via Interferon-β from Human Dendritic Cells
Source: Front Immunol. 2018 Jan 23;9:27. doi: 10.3389/fimmu.2018.00027 (PMC5787129; doi:10.3389/fimmu.2018.00027)
Supplement: Supplementary file 1 [file Data_Sheet_1.docx]

Supplementary Information

**Double-stranded RNA derived from lactic acid bacteria augments Th1 immunity via interferon-β from human dendritic cells**

Tadaomi Kawashima^a,b*^, Naho Ikari^a,b^, Yohei Watanabe^b^, Yoshiro Kubota^c^, Sachiyo Yoshio^d^, Tatsuya Kanto^d^, Shinichiro Motohashi^e^, Naoki Shimojo^f^, Noriko M Tsuji^b*^

^a^ Research and Development Division, Kikkoman Corporation, Chiba, Japan

^b^ Biomedical Research Institute, National Institute for Advanced Industrial Science and Technology (AIST), Tsukuba, Japan

^c^ Kikkoman General Hospital, Kikkoman Corporation, Chiba, Japan

^d^ The Research Center for Hepatitis and Immunology, National Center for Global Health and Medicine, Chiba, Japan

^e^ Department of Medical Immunology, Graduate School of Medicine, Chiba University, Chiba, Japan

^f^ Department of Pediatrics, Graduate School of Medicine, Chiba University, Chiba, Japan

* CORRESPONDENCE:

Dr. Tadaomi Kawashima, takawashima@mail.kikkoman.co.jp

Dr. Noriko M. Tsuji, nm-tsuji@aist.go.jp

Supplementary Table

Table S1 Cytokine production by CD1a^+^ and CD1a^−^ moDCs from 5 donors in stimulation with heat-killed K15 for 24 h.

|  |  | Donor No. | | | | |  |  |  |
| --- | --- | --- | --- | --- | --- | --- | --- | --- | --- |
|  |  | #1 | #2 | #3 | #4 | #5 | Ave | SD | t-test |
| IL-12  (pg/mL) | CD1a^+^ | 38.0 | 357.0 | 40.8 | 156.9 | 519.4 | 22.4 | 210.7 | 0.088 |
|  | CD1a^−^ | ND | 96.7 | ND | 15.0 | 66.4 | 35.6 | 43.7 |  |
| IL-10  (pg/mL) | CD1a^+^ | 438.2 | 200.0 | 58.4 | 238.3 | 70.9 | 210.2 | 171.7 | 0.427 |
|  | CD1a^−^ | 406.9 | 1400.0 | 203.2 | 59.3 | 77.5 | 429.4 | 560.0 |  |
| IL-6  (ng/mL) | CD1a^+^ | 8.8 | 2.4 | ND | 0.7 | 1.3 | 2.6 | 3.6 | 0.155 |
|  | CD1a^−^ | 14.8 | 118.7 | 50.2 | 0.6 | 3.2 | 37.5 | 49.5 |  |

(ND: not detected)

Supplementary Figure Legends

**Figure S1.** (A) PBMCs from the other donor in Figure 1B were cultured in medium alone (−) or stimulated with untreated or RNase A-treated heat-killed LAB strains for 24 h. Tested strains are described in Table 1. IL-12 concentration in culture medium was quantified by ELISA. Data are the mean ± SD of triplicates. **p*<0.05, ***p*<0.01 (Student’s *t*-test). (B) dsRNA in LAB are not involved in IL-6 and IL-10 secretion from PBMCs. PBMCs were cultured in medium alone (−) or stimulated with untreated or RNase A-treated heat-killed LAB strains for 24 h. Tested strains are described in Table 1. IL-10 and IL-6 concentrations in culture medium were quantified by ELISA. Data are the mean ± SD of triplicates and are representative of two different donors. (C) PBMCs from the other donor in Figure 1D were cultured in medium alone (−) or stimulated with heat-killed K15 or *Bacteroides* sp. for 24 h. Tested strains are described in Table 1. IL-12 concentration in culture medium was quantified by ELISA. Data are the mean ± SD of triplicates (ND: not detected). (D) PBMCs from the other donor in Figure 1E were cultured in medium alone (−) or stimulated with heat-killed K15 in the presence or absence of 20 μg/ml anti-human IFN-β mAb (αIFN-β) for 24 h. Mouse IgG1 Ab was used as the isotype control (Cont Ab). IL-12, IL-10 and IL-6 concentrations in culture medium were quantified by ELISA. Data are the mean ± SD of triplicates. ***p*<0.01 (Student’s *t*-test). The figure is related to Figure 1.

**Figure S2.** (A) moDCs from the other donor in Figure 2A were cultured in medium alone (−) or stimulated with heat-killed K15 in the presence or absence of 20 μg/ml anti-human IFN-β mAb (αIFN-β) for 24 h. Mouse IgG1 Ab was used as the isotype control (Cont Ab). IL-12, IL-10 and IL-6 concentrations in culture medium were quantified by ELISA. Data are the mean ± SD of triplicates. **p*<0.05 (Student’s *t*-test). (B) moDCs were stained with FITC-conjugated CD11c, BV421-conjugated CD14, BV605-conjugated HLA-DR and PE-conjugated CD1a. CD1a^+^ and CD1a^−^ moDCs were sorted from CD11c^+^CD14^−^HLA-DR^+^ cells. (C) moDCs from the other donor in Figure 2B were cultured in medium alone (−) or stimulated with untreated or RNase A-treated heat-killed K15 for 24 h. IL-12, IL-10 and IL-6 concentrations in culture medium were quantified by ELISA. Data are the mean ± SD of triplicates. ***p*<0.01 (vs untreated K15, Student’s *t*-test). (D) moDCs from the other donor in Figure 2C were cultured in medium alone (−) or stimulated with untreated or RNase A-treated heat-killed K15 for 9 h. Levels of IFN-β, IL-12 and IRF mRNA expressions were determined by quantitative RT-PCR. Expression is represented as relative expression compared with unstimulated moDCs. Data are the mean ± SD of triplicates. **p*<0.05, ***p*<0.01 (vs untreated K15, Student’s *t*-test). The figure is related to Figure 2.

**Figure S3.** moDCs from the other donor in Figure 3 were cultured in medium alone (−) or stimulated with heat-killed K15, *Bacteroides* sp., LPS or poly(I:C) in the presence or absence of chloroquine (5 μM) for 24 h. IL-12, IL-10 and IL-6 concentrations in culture medium were quantified by ELISA. Data are the mean ± SD of triplicates. **p*<0.05, ***p*<0.01 (Student’s *t*-test). The figure is related to Figure 3.

**Figure S4.** (A) BDCA1^+^ DCs (mDC1) from the other donor in Figure 4A were cultured in medium alone (−) or stimulated with untreated or RNase A-treated (0 M or 0.3 M NaCl) heat-killed K15 for 24 h. IL-12 concentrations in culture medium were quantified by ELISA. Data are the mean ± SD of triplicates. ***p*<0.01 (vs untreated K15, Student’s *t*-test). (B,C) LAB stimulation enhances HLA-DR and CD86 expression of BDCA1^+^ DCs. BDCA1^+^ DCs (mDC1) isolated from PBMCs were cultured in medium alone (−) or stimulated with heat-killed K15 for 24 h. After co-culture, cells were collected and stained with BV605-conjugated HLA-DR (B) and APC-conjugated CD86 (C). Data are represented as a histogram and mean fluorescence intensity (MFI) ± SD of triplicates, and are representative of two different donors. (D,E) Naïve CD4^+^ T cells purified from the other donor in Figure 4B and 4C were stimulated with mDC1 in the absence (−) or in the presence of untreated or RNase A treated (0 M or 0.3 M NaCl) K15 under neutral conditions containing only IL-2 (D) or Th2 conditions containing IL-2, IL-4 and anti-IFN-γ mAb (E). After 7 days, the percentage of cells producing cytoplasmic IFN-γ and IL-4 was determined by flow cytometry. Numbers indicate the percentage of the total cells present in each quadrant. (F) PBMCs from the other donor in Figure 4D were cultured in medium alone (−) or stimulated with heat-killed K15 in the presence or absence of 20 μg/ml anti-human IFN-β mAb (αIFN-β) for 5 days. IFN-γ, IL-4 and IL-17 concentrations in culture medium were quantified by ELISA. Data are the mean ± SD of triplicates (ND: not detected). **p*<0.05 (Student’s *t*-test). The figure is related to Figure 4.

Supplementary Figures

Figure S1 Kawashima *et al.*

Figure S2 Kawashima *et al.*

Figure S3 Kawashima *et al.*

Figure S4 Kawashima *et al.*
